# Early-Life Racial Discrimination, Racial Centrality, and Adult Allostatic Load Among African American Older Adults

**DOI:** 10.1093/geroni/igab046.719

**Published:** 2021-12-17

**Authors:** Courtney Thomas Tobin, Angela Gutierrez, Roland Thorpe

**Affiliations:** 1 Fielding School of Public Health, University of California Los Angeles, Fielding School of Public Health UCLA, California, United States; 2 Edward R. Roybal Institute on Aging, Los Angeles, California, United States; 3 Johns Hopkins Bloomberg School of Public Health, Baltimore, Maryland, United States

## Abstract

This study evaluated the life course processes through which early life racial discrimination (ELRD) and racial centrality (i.e., the importance of Black identity to one’s sense of self) interact to shape allostatic load (AL) among African American (AA) adults aged 50+ in the Nashville Stress and Health Study (N=260). Adolescent ELRD was associated with greater racial centrality in adulthood and conferred 35% greater risk of high adult AL; greater centrality was also linked to high adult AL. Centrality accounts for 24% of the association between ELRD and AL. ELRD and centrality interact to shape adult AL, such that racial centrality is protective against high AL for adults who experienced racial discrimination as children or adolescents. Findings highlight the multiple pathways through which race-related stressors and psychosocial resources interact to shape physiological dysregulation in later life and underscore the health significance of racial identity for older AAs.

